# Dataset on the safety behavior among Pakistani healthcare workers during COVID-19

**DOI:** 10.1016/j.dib.2022.107831

**Published:** 2022-01-19

**Authors:** Muhammad Awais-E-Yazdan, Muhammad Awais Ilyas, Muhammad Qamar Aziz, Muhammad Waqas

**Affiliations:** a College of Business, Universiti Utara Malaysia (UUM), Kedah Darul Aman, Sintok 06010, Malaysia; b Lahore Business School, The University of Lahore, Lahore, Punjab, Pakistan; c Universiti Teknologi MARA, Jalan Ilmu 1/1, Shah Alam, Selangor 40450, Malaysia; d Lahore Business School, The University of Lahore, Lahore, Punjab, Pakistan

**Keywords:** Safety compliance, Workplace safety, Safety management, Leadership styles

## Abstract

The dataset includes the particulars of 515 respondents on safety behavior during COVID-19. The questionnaires were adapted using Social Learning Theory and Social Exchange Theory. The variables included in dataset are Transactional Leadership (TSL), Transformational Leadership (TFL), Employee Well-Being (EWB) and Safety behavior (SB). Moreover, the dataset also contains the demographic profile of the respondents. Data was collected with the help of self-administered questionnaire from eight public hospitals in Punjab, Pakistan, namely Services Hospital Lahore, Sir Ganga Ram Hospital Lahore, Government General Hospital Faisalabad, DHQ Hospital Chiniot, Municipal General Hospital Sargodha, DHQ Hospital Jhang, DHQ Hospital Multan and Sulehri Children & General Hospital Sialkot. This dataset could provide a significant insight for future research in employee safety behavior.

## Specifications Table

**Table:** 

Subject	Organizational behavior
Specific subject area	Human resource management
Type of data	Table
How data was acquired	Data was collected through self-administered questionnaire using five point likert scale. The data also includes demographic features of respondents.
Data format	Raw, analyzed
Description of data collection	Data was collected via self-administered questionnaire in Punjab province. Total 550 questionnaires were distributed and after removing incomplete questionnaires only 515 were used in data analysis. It took 3 months for data collection.
Data source location	Hospitals: Services Hospital Lahore, Sir Ganga Ram Hospital Lahore, Government General Hospital Faisalabad, DHQ Hospital Chiniot, Municipal General Hospital Sargodha, DHQ Hospital Jhang, DHQ Hospital Multan and Sulehri Children & General Hospital Sialkot. Country: Pakistan
Data Accessibility	Repository name: Mendeley data Data identification number: Direct URL to data: https://data.mendeley.com/datasets/wt2dbjcgc8/draft?a=703cfd69-8e84-47f1-884f-8df3e61a0774

## Value of the Data

• The dataset provide the evidence that organizations should implement workplace policies and procedures that influence safety behavior.
• The dataset is useful to address other related issues. For instance: safety climate, safety culture and safety citizenship behavior.
• The dataset is helpful to predict the attitude and behavior of employees at early stage of employment.
• The dataset focused on the hurdles which slow down the leadership practices in order to achieve a safe work environment.
• The dataset advocates that management must invest to satisfy the psychological needs of the workers to shine their basic workplace skills.
• The dataset could be used by other researchers in order to compare this data with other data obtain from related studies but different geographic regions.

## 1 Data Description

The dataset includes the questions related to four constructs:

Transactional leadership (TSL), Transformational leadership (TFL), Employee well-being (EWB) and Safety behavior (SB). Definitions of all constructs and references relating to instrument are given in Table 1.

**Table 1:** Variables, code, definition and reference of each instrument.

Variable	Code	Definition	References of the instrument
Transactional leadership	TSL	It refers to an exchange process where leaders and followers exchange valuable information with each other.	[2,3]
Transformational leadership	TFL	A type of leadership which morally and ethically support both leaders and their followers in a mutual consent.	1,3]
Employee well-being	EWB	Refers to the physical (tiredness, muscular pain & headache) and mental (anxiety, self-respect & depression) factors of the individuals.	[4,5]
Safety behavior	SB	It refers to maintain the safe working standard by wearing personal protective equipment.	[6,7]

The SPSS sheet along with questionnaire are given as a supplementary file. The questionnaires for TSL and TFL were adapted [3] to explain the relationship by incorporating social learning theory. Similarly, the questionnaires for SB and EWB are also adapted [5,7]. The relationship between EWB and SB were explained by social exchange theory. Five-point Likert scale ranging (1= strongly dis-agree, 2= dis-agree, 3= neutral, 4= agree, 5= strongly agree) was incorporated which enhance the quality of responses and lower the tiredness of the respondents. TSL consist of five items, whereas TFL consist of six items. Items for TSL and TFL were adapted from previous study [3]. In addition, items for EWB and SB were adapted from different studies [5,7] with three and seven items respectively. Structural Equation Modeling technique using smart PLS 3.2.6 has been incorporated to explain the measurement and structural model.

## 2 Materials and Methods

Data was gathered through convenience sampling. It is a type of non-probability technique in which the data is collected from the people easily to approach. Data is collected from the public hospitals of Punjab Pakistan. Province Punjab is selected for data collection as it is the second most populous province and is known for quality hospitals. Data was entered and coded in SPSS software. All the preliminary tests were conducted on SPSS. For the main analysis Smart PLS 3.2.6 was used.

**Table 2:** Loadings, average variance extracted and composite reliability.

Constructs	Items	Loadings	AVE	CR
Transactional leadership	TSL1	0.728	0.552	0.860
	TSL2	0.729		
	TSL3	0.813		
	TSL4	0.753		
	TSL5	0.688		
Transformational leadership	TFL1	0.587	0.515	0.863
	TFL2	0.796		
	TFL3	0.793		
	TFL4	0.744		
	TFL5	0.713		
	TFL6	0.649		
Employee well-being	EWB1	0.820	0.655	0.850
	EWB2	0.801		
	EWB3	0.806		
Safety compliance	SC1	0.658	0.514	0.863
	SC3	0.819		
	SC4	0.786		
	SC5	0.725		
	SC6	0.661		
	SC7	0.633		

*Note:* AVE = Average Variance Extracted, CR = Composite Reliability.

### 2.1 Loadings, composite reliability and average variance extracted

Individual item reliability of construct above 0.30 can be retained [8]. Similarly, items must be removed if the removal increase the value of average variance extracted (AVE) and composite reliability (CR). Therefore, the present dataset removed one item (SC2) as the removal increase the value of AVE and CR. In addition, the value of AVE is above than 0.5 which means convergent validity is established.

Table 2 shows the adequate, individual item reliability, CR and AVE of the study's constructs.

Discriminant validity is considered valid as the values in Tables 3, 4 and 5 were in acceptable range.

**Table 3:** Latent variable correlations and square roots of (AVE).

	EWB	SC	TFL	TSL
EWB	**0.809**			
SC	0.314	**0.717**		
TFL	0.457	0.612	**0.718**	
TSL	0.258	0.656	0.673	**0.743**

*Note:* Entries in the boldface represent the square root of average variance extracted (AVE).

**Table 4:** Cross loadings.

	EWB	SC	TFL	TSL
**EWB1**	**0.820**	0.294	0.488	0.284
**EWB2**	**0.801**	0.179	0.328	0.176
**EWB3**	**0.806**	0.261	0.266	0.147
**SC1**	0.384	**0.658**	0.515	0.376
**SC3**	0.293	**0.819**	0.511	0.513
**SC4**	0.245	**0.786**	0.482	0.450
**SC5**	0.097	**0.725**	0.389	0.545
**SC6**	0.128	**0.661**	0.337	0.410
**SC7**	0.186	**0.633**	0.378	0.510
**TFL1**	0.227	0.430	**0.587**	0.561
**TFL2**	0.287	0.549	**0.796**	0.639
**TFL3**	0.296	0.428	**0.793**	0.642
**TFL4**	0.415	0.440	**0.744**	0.380
**TFL5**	0.406	0.421	**0.713**	0.327
**TFL6**	0.366	0.317	**0.649**	0.256
**TSL1**	0.170	0.491	0.394	**0.728**
**TSL2**	0.203	0.497	0.418	**0.729**
**TSL3**	0.232	0.565	0.664	**0.813**
**TSL4**	0.149	0.426	0.534	**0.753**
**TSL5**	0.195	0.440	0.476	**0.688**

**Table 5:** HTMT correlation matrix for discriminant validity.

	EWB	SC	TFL	TSL
**EWB**	-			
**SC**	0.385	-		
**TFL**	0.583	0.741	-	
**TSL**	0.322	0.810	0.808	-

## Ethics Statement

It is stated that the consent was taken from each individual who participated in this survey.

## CRediT authorship contribution statement

**Muhammad Awais-E-Yazdan:** Conceptualization, Data curation, Writing – review & editing, Methodology. **Muhammad Awais Ilyas:** Visualization, Software. **Muhammad Qamar Aziz:** Investigation, Validation. **Muhammad Waqas:** Supervision.

## Declaration of Competing Interest

The authors declared that there is no conflict of interest either financial or personal among them.

## Appendix Supplementary materials

Image, application 1

Image, application 2

Image, application 3
