# Supplementary material for: Dataset on the safety behavior among Pakistani healthcare workers during COVID-19
Source: Data Brief. 2022 Jan 19;41:107831. doi: 10.1016/j.dib.2022.107831 (PMC8767930; doi:10.1016/j.dib.2022.107831)
Supplement: Supplementary file 1 [file mmc1.docx]

**SECTION A: DEMOGRAPHIC INFORMATION**

**Kindly tick mark the appropriate boxes and fill the blanks according to the questions below.**

1. Gender:

❒ Male

❒ Female

1. Age (in years)

1. Job title
2. Marital Status

❒Single

❒Married

❒Divorced

❒Widowed

1. How long have you been working with the current company (years)

**SECTION B – TRANSACTION & TRANSFORMATIONAL LEADERSHIP**

The following questions are related to transactional & transformational leadership. For each statement, please, tick mark the number that indicates the extent to which you agree or disagree with the statement.

|  | **Questions** | **Strongly**  **Disagree** | **Disagree** | **Neutral** | **Agree** | **Strongly Agree** |
| --- | --- | --- | --- | --- | --- | --- |
| 1. | Makes clear expectation | 1 | 2 | 3 | 4 | 5 |
| 2. | Will take action before problems are chronic | 1 | 2 | 3 | 4 | 5 |
| 3. | Tells us standards to carry out work | 1 | 2 | 3 | 4 | 5 |
| 4. | Works out agreements with me | 1 | 2 | 3 | 4 | 5 |
| 5. | Monitors my performance and keeps track of mistake | 1 | 2 | 3 | 4 | 5 |
| 6. | Listens to my concerns | 1 | 2 | 3 | 4 | 5 |
| 7. | Encourages me to perform | 1 | 2 | 3 | 4 | 5 |
| 8. | Increases my motivation | 1 | 2 | 3 | 4 | 5 |
| 9. | Encourages me to think more creatively | 1 | 2 | 3 | 4 | 5 |
| 10. | Sets challenging standards | 1 | 2 | 3 | 4 | 5 |
| 11. | Gets me to rethink never-questioned ideas | 1 | 2 | 3 | 4 | 5 |

**SECTION C –EMPLOYEE WELL-BEING**

The following questions are related to employee well-being. For each statement, please, tick mark the number that indicates the extent to which you agree or disagree with the statement.

|  | **Questions** | **Strongly**  **Disagree** | **Disagree** | **Neutral** | **Agree** | **Strongly Agree** |
| --- | --- | --- | --- | --- | --- | --- |
| 1. | How would you rate your quality of life? | 1 | 2 | 3 | 4 | 5 |
| 2. | How satisfied are you with yourself? | 1 | 2 | 3 | 4 | 5 |
| 3. | How satisfied are you with your capacity for work? | 1 | 2 | 3 | 4 | 5 |

**SECTION D - SAFETY BEHAVIOR**

The following questions are related to safety behavior. For each statement, please, tick mark the number that indicates the extent to which you agree or disagree with the statement.

|  | **Questions** | **Strongly**  **Disagree** | **Disagree** | **Neutral** | **Agree** | **Strongly Agree** |
| --- | --- | --- | --- | --- | --- | --- |
| 1. | I use all necessary safety equipments to do my job | 1 | 2 | 3 | 4 | 5 |
| 2. | I carry out my work in a safe manner | 1 | 2 | 3 | 4 | 5 |
| 3. | I follow correct safety rules and procedures while carrying out my job | 1 | 2 | 3 | 4 | 5 |
| 4. | I ensure the highest levels of safety when I carry out my job | 1 | 2 | 3 | 4 | 5 |
| 5. | Occasionally due to lack of time, I deviate from correct and safe work procedures | 1 | 2 | 3 | 4 | 5 |
| 6. | Occasionally due to over familiarity with the job, I deviate from correct and safe work procedures | 1 | 2 | 3 | 4 | 5 |
| 7. | It is not always practical to follow all safety rules and procedures while doing a job | 1 | 2 | 3 | 4 | 5 |
